# TET2 deficiency promotes anxiety and depression-like behaviors by activating NLRP3/IL-1β pathway in microglia of allergic rhinitis mice

**DOI:** 10.1186/s10020-023-00757-9

**Published:** 2023-11-27

**Authors:** Ziang Gao, Hao Lv, Yunfei Wang, Yulie Xie, Mengting Guan, Yu Xu

**Affiliations:** 1 Department of Otolaryngology-Head and Neck Surgery, Renmin Hospital of Wuhan University, Wuhan, 430060 China; 2 Department of Otolaryngology-Head and Neck Surgery, The First Affiliated Hospital of Soochow Hospital, Suzhou, 215000 China; 3 Institute of Otolaryngology Head and Neck Surgery, Renmin Hospital of Wuhan University, Wuhan, China; 4 Hubei Province Key Laboratory of Allergy and Immunology, Wuhan University, Wuhan, 430060 China

**Keywords:** Allergic rhinitis, Anxiety and depression-like behavior, TET2, NLRP3/IL-1β, Microglia, Metformin

## Abstract

**Background:**

Anxiety and depression-like behaviors in allergic rhinitis (AR) are attracting attention, while the precise mechanism has not been clearly elucidated. Recent evidence shows that neuroinflammation in anterior cingulate cortex (ACC) may be the core of these neuropsychiatric symptoms in AR. Here, we investigated the molecular link between the anxiety and depression-like behaviors and neuroinflammation in ACC.

**Methods:**

Mice were sensitized and challenged with ovalbumin (OVA) to induce AR. Nasal inflammation levels were assessed by H&E staining and PAS staining. Anxiety and depression-like behaviors were evaluated by behavioral experiments including open field test, forced swimming test, and sucrose preference test. Neuronal impairment was characterized via Nissl staining and ^18^FDG-PET. The role of ten-eleven translocation 2 (TET2) in AR-related anxiety and depression was assessed by *Tet2−/−* mice. In addition, the murine BV2 microglial cell line was utilized to explore the molecular mechanisms by which TET2 mediates neuroinflammation. The levels of TET2, NLRP3 and their downstream molecules were detected by immunohistochemistry, Western blot, Dot blot and ELISA. The effects of metformin on depression-like behaviors in AR mice were also evaluated.

**Results:**

AR mice showed significant anxiety and depression-like behaviors, which associated with the activation of ACC. Loss of TET2 activated the NLRP3/IL-1β pathway of microglia in AR mice, further accelerating the anxiety and depression-like behaviors. In addition, knockdown of TET2 activated the NLRP3/IL-1β pathway in BV2 cells. Metformin improved the neuropsychiatric symptoms of AR mice by reducing the activation of NLRP3/IL-1β pathway after upregulating TET2.

**Conclusion:**

TET2 deficiency activates the NLRP3/IL-1β pathway of microglia in the ACC, promoting the pathological process of anxiety and depression-like behavior in AR. Metformin could be effective in treating neuroinflammation by regulating microglia via TET2 up-regulation, indicating that metformin is a potential way to treat anxiety and depression-like behaviors in AR.

## Introduction

Allergic rhinitis (AR) is a chronic nasal inflammatory disease characterized by IgE responses and the development of type 2 immunity (Zhong et al. 2017). The classic symptoms include sneezing, nasal itching, nasal congestion, and rhinorrhea, which severely affect the patient's quality of life (Greiner et al. 2011). AR affects 10–40% of the general population worldwide. More worryingly, the prevalence of AR has risen at an alarming rate over the past few decades (Brożek et al. 2017). It is clear that AR is becoming a global medical, public health, and economic issue.

Anxiety and depression are common mental health concerns in children, adolescents and adults, which are among the major contributors to the global health burden (Tiller 2013). A growing number of epidemiological and clinical evidences have revealed a close correlation between AR and high risk of depression and anxiety (Rodrigues et al. 2021; Bedolla-Barajas et al. 2017). Cuffel et al. analyzed 85,000 subjects and found that anxiety symptoms were 1.4 times higher depression symptoms were 1.8 times higher in individuals with AR compared to healthy controls (Cuffel et al. 1999). A recent large Spanish cohort study also showed a significant effect of AR on anxiety and depression symptoms (Muñoz-Cano et al. 2018). And AR patients with severe and perennial symptoms appear to suffer more strongly from effects of anxiety and depression (Muñoz-Cano et al. 2018). The emotional, psychological, memory and cognitive problems of AR patients suggest the existence of brain symptoms, but the underlying mechanism is unclear. The primary reason for this is that mechanisms of depression and anxiety in AR is not well understood. We will study the anxiety and depression-like behavior of AR from the molecular biological level.

In our previously published study, by comparing AR patients with healthy people, we found that AR patients were more likely to exhibit anxiety and depression-like symptoms and behaviors, and fMRI results suggested that this change was probably caused by the activation of the anterior cingulate cortex (ACC) (Gao et al. 2021). We found that the severity of anxiety and depression, nasal symptoms, and serum specific IgE levels were correlated with the degree of activation in the ACC. The ACC is a brain region involved in the processing of negative emotions and a key hub of pain-induced depression (Tolomeo et al. 2016). Neuroinflammatory remodeling in the ACC is also known to be a key driver of anxiety, depression, and cognitive dysfunction in GI disorders (Matisz and Gruber 2022). Therefore, we hypothesized that the anxiety-depression-like behaviors of AR patients are closely related to the functional changes of ACC.

In another study, we found that Ten-eleven translocation 2 (TET2) plays a very important role in the occurrence and development of allergic rhinitis. TET protein is a dioxygenase, and TET2 is a member of the TET family. Studies have found that the reduction of 5-hmC is associated with anxiety-like behaviors (Antunes et al. 2021), in which TET2 plays a key role. In the studies related to depression and depression-like behaviors, the low expression of TET2 also leads to the aggravation of disease symptoms (Ji et al. 2021). Neurodegenerative disease related studies have revealed that TET2 reduction exacerbates Alzheimer's disease amyloid burden and cognitive impairment (Li et al. 2021). In addition, studies have shown that TET2 is associated with microglial inflammatory response (Carrillo-Jimenez et al. 2019). Therefore, we hypothesized that in AR, TET2 may regulate the neuroimmune pathway by affecting the microglia in the ACC, leading to the anxiety and depression related symptoms of AR.

Neuroinflammation caused by microglial activation is also thought to induce anxiety and depression-like behaviors. This speculation has been directly verified in chronic stress models (Wang et al. 2018). A growing body of research suggests that neuroinflammation plays a key role in the development of anxiety or depression like behaviors. Microglia are important immune effector cells in the CNS, which play a continuous role in immune surveillance of the CNS, and they share a common embryological origin with peripheral macrophages. Microglia can interact with many other cells in the CNS, including neurons, astrocytes, and oligodendrocytes.

NLRP3 inflammasome is a multiprotein complex abundantly expressed in microglia of the central nervous system. It is composed of the cytosolic receptor NLRP3, the adaptor protein apoptosis-associated speck-like protein (ASC) and the precursor of the effector protein caspase-1. The current study identifies the NLRP3 inflammasome as a key factor in the development of neuroinflammation. NLRP3 inflammasome can widely sense a variety of endogenous and exogenous stimuli, leading to caspase-1 activation, which further promotes the maturation and release of IL-1β and IL-18. However, IL-1β and IL-18 can play an important role in inducing and maintaining neuroinflammation by regulating synaptic plasticity (He et al. 2019).

In the central nervous system, metformin can improve the development and progression of neuroinflammation by reducing the activation of microglia. Metformin is a classic treatment for type 2 diabetes. In addition to its hypoglycemic effect, metformin also has therapeutic effects on central nervous system diseases. Previous studies have shown that metformin may be a good drug choice for patients with co-existing symptoms of anxiety and depression (Jiang et al. 2014). Besides, metformin reduces depression-like behavior induced by chronic stress in mice (Fang et al. 2020). It has been shown that metformin inhibits NLRP3 activation in other disease models (Jia et al. 2020; Xian et al. 2021). Wu et al found that metformin treatment increased TET2 stability and 5-hmC levels (Wu et al. 2018). This article will investigate whether metformin can improve AR neuroinflammatory symptoms through TET2.

Taken together, we hypothesized that anxiety and depression in AR are associated with the presence of microglia-mediated inflammation in the ACC, which is partly due to dysregulation of the TET2/NLRP3 axis in microglia. We constructed an animal model of AR using wild-type mice to verify the relationship between neuroinflammation in the prefrontal cortex and anxiety and depression in AR, and explored the role of TET2-mediated NLRP3 activation in anxiety and depression in AR using *Tet2* knockout mice and in vitro experiments.

## Material and methods

### Mice

Six- to eight-week-old female C57BL/6 mice (20–25 g) were purchased from the Experimental Animal Center of Wuhan University (Wuhan, Hubei, China). Mice were housed in a specific-pathogen-free isolation environment in the Animal Experimental Center of the Renmin Hospital of Wuhan University. Standard laboratory conditions include a controlled temperature (23 ± 1 °C), a 12-h light/dark cycle, a moderate humidity (50–60%) and free access to water and food. All animals were acclimated for 1 week prior to the experiment. *Tet2*-deficient B6(Cg)-*Tet2*^tm1.2Rao^/J mice were obtained from The Jackson Laboratory (West Grove, PA, USA). Wild type (WT) controls and homozygous (*Tet2* knockout [*Tet2*^−/−^]) mice were bred from heterozygous parents and all mice were genetically identified as described previously (Tan et al. 2022).

### Mouse model of OVA‐induced AR

**Fig. 1:**
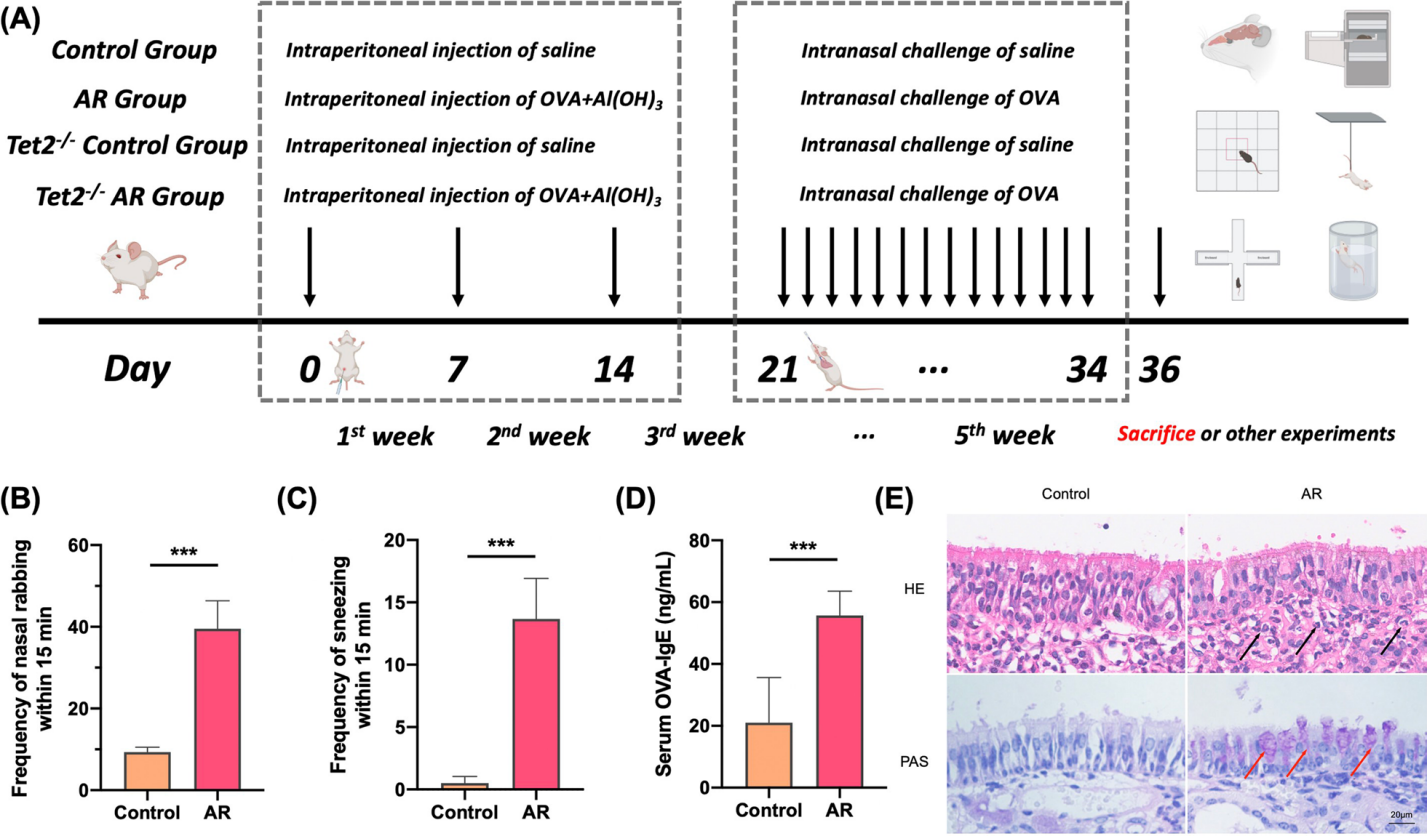
Flow chart of mouse model and confirmation of successful establishment of AR model. **A** In AR group, 300 μL OVA was intraperitoneally injected at 0, 7 and 14 days after the start of the experiment to achieve basal sensitization. On days 21 to 35 of the experiment, local excitation was performed by intranasal infusion of 20 μL OVA nasal drops at 2 PM every day. On the 36th day of the experiment, behavioral experiments were prepared or sacrificed. Mice in control group were treated with normal saline instead of OVA intraperitoneal injection and OVA nasal drops. The model of Tet2−/− control group was the same as that of control group. Mice in Tet2−/−AR group were modeled in the same way as those in AR group. **B** Quantitative analysis of sneezing times in control group and AR group within 15 min. **C** Quantitative analysis of nasal scratching times within 15 min in control group and AR group. **D** Comparison of serum OVA-specific IgE levels between control group and AR group. **E** HE and PAS staining of mouse nasal mucosa (×400 times). Black arrows indicate eosinophils and red arrows indicate goblet cells. (***p < 0.001)

The mice were randomly divided into a control group (WT control group), an allergic rhinitis group (WT AR group), a *Tet2*^−/−^ control group (*Tet2*^−/−^ control group) and a *Tet2*^−/−^ allergic rhinitis group (*Tet2*^−/−^ AR group) each consisting of 10 mice. In the AR and *Tet2*^−/−^ AR group, mice were sensitized by an intraperitoneal administration of 100 μg ovalbumin (OVA, grade V; Sigma-Aldrich, St. Louis, MO, USA) and 5 mg aluminum hydroxide in 300 μL normal saline on days 0, 7, and 14. The control group received an intraperitoneal injection of the same dose of saline. The mice were then intranasally challenged with 20 μL saline (10 μL per nostril) containing 200 μg OVA daily for 2 weeks starting on day 14. Similarly, in the control group, mice were intranasally challenged with the same amount of saline. In AR + Met group, in the local challenge stage, the metformin solution (250 mg/kg) was injected intraperitoneally to remove endotoxin 30 min before each challenge, and the other mice were the same as the AR group. Within 15 min after the last OVA challenge, the frequency of nasal rubbing and sneezing in each mouse was counted to quantitatively evaluate the symptoms of AR. Figure 1 provides a summary of the experimental procedure.

### Measurement of OVA‐specific IgE

Serum levels of OVA-specific IgE were determined using a Mouse OVA-specific IgE ELISA Kit (Cayman, Ann Arbor, USA) according to the manufacturer's instructions.

### Hematoxylin and Eosin (HE) staining

Mice were anesthetized with 3% isoflurane inhalation and quickly decapitated. Coronal brain slices (300 µm) containing ACC were prepared by standard methods and tissues of bilateral ACC were dissected in ice-cold artificial cerebrospinal fluid (ACSF) under the anatomical microscope (Xu et al. 2008). Noses and ACC tissues were fixed in 4% paraformaldehyde for 48 h. Additionally, the noses were decalcified in decalcified solution for 2 weeks and then made into paraffin sections. After the paraffin sections were deparaffinized, hematoxylin was used to stain the nucleus and eosin was used to stain the cytoplasm. The morphology of the nasal mucosa and ACC tissues were then observed under a light microscope (×400). Five fields on each slice were randomly selected, in which the number of eosinophils was counted under the microscope and then averaged.

### Periodic acid-Schiff (PAS) staining

The nasal mucosal tissues were fixed in a 4% paraformaldehyde solution. Then, the paraffin-embedded tissue samples were cut into 4-μm-thick sections. The sections were stained with periodic acid-Schiff (PAS) stain for goblet cells. The numbers of these cells were counted in 4 randomly high-power fields (magnification 400×).

### Nissl staining

Mice were perfused transcardially with 200 mL of ice-cold phosphate-buffered saline, followed by 300 mL of phosphate-buffered 10% formalin. After perfusion, the brain was fixed for 48 h, dehydrated, and embedded in paraffin. Hippocampal tissues were collected prepared as 10-μm-thick sections and stained by a Nissl dye. The experiment was performed using a Nissl staining kit (Solarbio, China) according to the manufacturer’s instructions. Nissl-stained cells in the ACC areas were observed at ×400 magnifications.

### Immunofluorescence staining

The ACC tissues were fixed with 4% paraformaldehyde, embedded in slices, and baked. After the paraffin sections were completely dewaxed with xylene, 10% calf serum was added, and the section were placed at room temperature for 10 min. The sections were incubated with anti-NLRP3 (1:500, Proteintech), anti-Caspase-1 (1:1000, Proteintech), anti-ASC (1:1000, ABclonal), and anti-IBA-1 (1:1000, Abcam) at 4 °C overnight, followed by the goat anti-mouse or goat anti-rabbit secondary antibody for 1 h at room temperature. The sections were washed with water, blown dry, sealed with glycerin, and followed by observation under a fluorescence microscope (Olympus, Tokyo, Japan). For cellular immunofluorescence, BV2s were seeded into 6-well plates containing a coverslip per well at a density of 0.25–1 × 10^6, fixed with 4% paraformaldehyde for 10 min and permeabilized with 0.2% Triton X-100 for 10 min. After being rinsed with PBS, cells were blocked with 2% BSA and then incubated with anti-NLRP3 (1:200, Proteintech), anti-Caspase-1 (1:200, Proteintech), anti-ASC (1:200, ABclonal), and anti-IBA-1 (1:1000, Abcam) at 4 °C overnight, followed by the goat anti-mouse or goat anti-rabbit secondary antibody for 1 h at room temperature. Expression of various indicators in different groups on three high-power fields (HPF, × 400) were studied by a fluorescence microscope (Olympus, Tokyo, Japan).

### Mouse PET experiment and software analysis procedure

Before PET imaging, mice were fasted for 12 h and then injected with approximately 200 ± 10 μCi of ^18^F-FDG (18-fluoro-6-deoxyglucose) through the tail vein. After 60 min of metabolism, mice were anesthetized with 2% isoflurane. TransPET Discoverist 180 (Suzhou Ray-can Technology Co., LTD) system was used for static scanning for 10 min to obtain images, and then CT images were scanned. PET images were reconstructed by (3D) OSEM with a 3D voxel size of 0.5 × 0.5 × 0.5 mm^3^, and CT images with a matrix of 256 × 256 × 256 were reconstructed by FDK algorithm. Region of interest (VoI) analysis was performed using Amide (Medical Imaging Data Review software) and Pmod (Pmod Technologies LLC, Zurich PET Center, Switzerland) software. The mean normalized uptake value (SUV) was calculated using the following formula: mean pixel value with decay corrected VoI (μCi/kg)/(injected dose [μCi]/ weight [kg]).

PET images were analyzed by software, ROIs were delineated, and SUV values of each ROI were calculated. The calculation method of SUV is shown in Eq. (1).

1 $$SUV = \frac{{C_{T} }}{{D_{Inj} }} \times \frac{{V_{T} }}{{W_{T} }} \times W_{S}$$

C_T_ is the activity in a unit volume of tissue. D_Inj_ is the injection dose. V_T_ is the volume of tissue. W_T_ is the mass of tissue. W_S_ is the mass of mice. Here, the tissue density V_T_/W_T_ is set to 1.

### Behavioral experiment and specific steps

#### Elevated cross maze experiment

The elevated cross maze mainly consists of a pair of open and closed arms that are perpendicular to each other and cross each other, connected in the middle. Data were collected by a surveillance camera located above the center of the experimental area, and processed by a computer animal behavior analysis software connected to the camera. The equipment size of mouse elevated cross maze: arm length 70 cm, arm width 5 cm, closed arm height 15 cm, arm height 50 cm off the ground.

Before the experiment, make sure that the whole device of the elevated cross maze is clean. In the experiment, the mice were gently removed from the cage with their backs to the experimenter as far as possible, and placed gently in the central area of the organ body, with the mice facing the open arm. Then the experimenter left quickly and quietly, clicked the start button of the software, and recorded the activity of the mice within 5 min. ETHOVISION animal movement trajectory tracking system was used to record and analyze the behavior of each mouse in the elevated cross maze experiment. Anxiety behavior was evaluated by the time the mice entered the open arm, and the longer the time they stayed in the closed arm, the more anxious the mice were. If any mouse remained stationary in the closed arm or fell from the open arm, the experimental data were excluded.

#### Open field test

The open field experimental apparatus consists of mouse open field experimental chamber and data acquisition and processing system. The specification of mouse experimental chamber is (length × width × height): 50 cm × 50 cm × 40 cm.

Before the experiment, it is necessary to ensure that the whole device of open field experiment is clean. In the experiment, the mice were gently removed from the cage as far away from the experimenter as possible and placed gently in the central area of the open field. Then the experimenter left quickly and quietly, clicked the start button of the software, and recorded the activity of the mice within 5 min. ETHOVISION animal motion trajectory tracking system was used to record and analyze the behavior of each mouse in the open field experiment. The total movement distance of mice was used to reflect the movement situation and reflect the spontaneous activity ability of mice. The anxiety and depression of mice were reflected by the residence time of central zone and the total times of entering central zone.

#### Tail suspension test

Remove the experimental animal gently from the cage, cut out 17 cm long electrical tape (1–1.5 cm wide), and mark 2 cm at one end. The marked part of the tape was connected to the tail of the mouse, leaving a distance of about 3 mm from the tail, and the remaining 15 cm of tape was used for hanging the mouse. Attach the tape to the hanging rod, so that the abdomen of the mouse faces the camera (to capture the movement of the limbs), and leave immediately; the activity of animals in the air was recorded for a total of 6 min. The first immobile time in the first 2 min and the accumulated immobile time in the next 4 min were recorded. The earlier the immobility time in the first 2 min, the more likely the animals were to induce depression. The longer the immobility time within the next 4 min, the more depressed the animals were.

#### Forced swimming test

Before the experiment, it is necessary to ensure that the whole device of the forced swimming experiment is clean. Then, tap water is added to the cylindrical water tank with a bottom radius of 6 cm and a height of 30 cm, the temperature is controlled at about 25 ℃ (the upper and lower levels are not more than 1 ℃), and the liquid level is adjusted to about 18 cm. In the experiment, mice were gently removed from their cages and quickly placed in water. At the same time, the activity of the mice was recorded for a total of 6 min. The cumulative immobility time of the animals after 4 min was counted. The longer the immobility time, the more serious the depression was. At the end of the experiment, the mice were put into the clean cage prepared in advance, and the animals were assisted to recover their body temperature. The next batch of experiments were conducted after changing water and cleaning.

#### Sucrose preference test

In brief, the mice were habituated to 1% sucrose solution for 1 h. Two bottles with 150 mL 1% sucrose solution or water were offered to mice. The bottles were weighted prior to or following the test. The mice were fed freely before the experiment, and tap water and sugar water consumption were measured by measuring the weight of the bottle. After calculating the consumption of water and sucrose solution, the formula was applied for determining the sucrose preference: sugar water preference value = sugar water consumption (g)/[sugar water consumption (g) + water consumption (g)] × 100%.

### Western blot analysis

The mouse tissues and BV2 cell lines were homogenized and lysed in RIPA buffer with added protease and phosphatase inhibitors to extract the total protein. The protein concentration was measured using a BCA Protein Assay Kit (Beyotime Biotechnology, China). Protein samples totaling 40 μg were added to each lane of the gel, separated on a 10% SDS-PAGE gel, and then transferred to a polyvinylidene fluoride (PVDF) membrane (Millipore) for 1.5 h at 200 mA. The PVDF membranes were sealed with 5% skim milk for 1 h at room temperature. The membranes were washed three times for 5 min each and then incubated with anti-TET2 antibody (1:1000, Proteintech), anti-NLRP3 (1:500, Proteintech), anti-Caspase-1 (1:1000, Proteintech), anti-ASC (1:1000, ABclonal), and anti-IBA-1 (1:1000, Abcam) antibody overnight at 4 °C. The membranes were washed three times with TBST for 10 min each and then incubated with the goat anti-mouse or goat anti-rabbit secondary antibody for 1 h at room temperature. The membranes were visualized by chemiluminescence using an Image Lab™ quantitative assay system (Bio-Rad, California, USA).

### Dot-blot assay

Levels of 5-hmC in the ACC were detected using dot-blot assays as described previously (Ko et al. 2010). Briefly, genomic DNA was isolated from brains of WT and AR mice. The DNA samples were denatured and dilution samples were spotted on nitrocellulose membranes. The blotted membrane was vacuum-baked at 70 °C for 1 h, blocked, and incubated with anti-5-hmC antibody (1:10000, CST) overnight at 4 °C. After being incubated secondary antibody, the membrane was visualized by Image Lab™ quantitative assay system (Bio-Rad, California, USA).

### Enzyme-linked immunosorbent assay (ELISA)

The ACC tissues, blood and culture media were collected after drug treatments. The ACC tissues on both sides of a mouse were taken, and 200 μL of lysate was added. After homogenization, the lysate was split on ice for 30 min and centrifuged at 12,000 rpm for 15 min. The protein concentration of the supernatant was measured by a BCA kit (Absin, Shanghai, China), and ensured that the total protein of each sample was 50 µg. Similarly, supernatants of blood and culture media were retained. The levels of inflammatory mediators were measured using ELISA kits of IL-1β, IL-18 (BD Biosciences, San Jose, CA, USA) and OVA-specific IgE (Bioswamp, Wuhan, China) according to the manufacturer's instructions, respectively. Briefly, 50 μL ELISA diluent and 50 μL sample were added to each well, and incubated at room temperature for 2 h. After aspirating and washing 5 times, 100 μL of the working detector was added and incubated at room temperature for 1 h. Then, 100 μL of TMB one-step substrate reagent was added and incubated at room temperature for 30 min. After adding 50 μL stop solution, read at 450 nm within 30 min. The experiments were repeated for 3 times.

### siRNA and plasmid transfection

BV2 microglia cells in culture flasks were seeded at a density of 5 × 10^5^ cells in each 6-well plate, shaken evenly, and gene knockdown could be carried out when the cell density reached about 30% under a light microscope. siRNAs against TET2 (5'-CTGCTTCTGTTCTCAATAA-3') were obtained from GenePharma Co. (Shanghai, China). siRNAs or plasmids were mixed with Lipofectamine 2000 (Invitrogen, Carlsbad, USA) in reduced serum medium (Opti-MEM; Gibco, USA) according to the manufacturer’s instructions. Transient transfections were carried out as described previously (Qin et al. 2022). The medium was replaced for the cells in the plate during the resting period. After 15 min, 200 μL mixture was added to each well, and 6-well plates were labeled, gently mixed, and placed in the incubator. After 24 h, the concentration and state of the cells were observed, and the fluorescence expression after transfection was observed under a fluorescence microscope if there was a fluorescent label. Then 1 mL of complete medium was added to each well to supply the cells with energy, and the cells were placed in an incubator for 12–24 h.

### Statistical analysis

All results are represented as mean ± standard error of mean. The data and graphs were analyzed by GraphPad Prism 8.0 (GraphPad Software, San Diego, CA, USA). The results were analyzed by 1-way analysis of variance followed by post hoc Tukey's tests for multiple comparisons. A P value of < 0.05 was considered significant.

## Results

### A mouse model of OVA‐induced AR was successfully established

The success of the AR model (Fig. 1A) in C57BL/6 mice was confirmed by observing symptoms, eosinophilic infiltration of nasal mucosa, and detection of serum OVA-specific IgE. The results showed that the mice in the AR group sneezed and scratched their noses significantly more often than the control mice within 15 min (Fig. 1B, C). In addition, the level of OVA-specific IgE was also significantly higher in the AR group than in the control group (Fig. 1D). Compared to the control mice, significant eosinophilic infiltration was observed in the nasal mucosa in the AR group (Fig. 1E). These results demonstrate that the mouse model of AR was successfully established.

### TET2 depletion aggravates anxiety and depression-like behaviors in AR mice

In order to confirm the occurrence of anxiety-depression-like behaviors in AR mice, the following four behavioral experiments were performed.

#### Elevated cross maze experiment

**Fig. 2:**
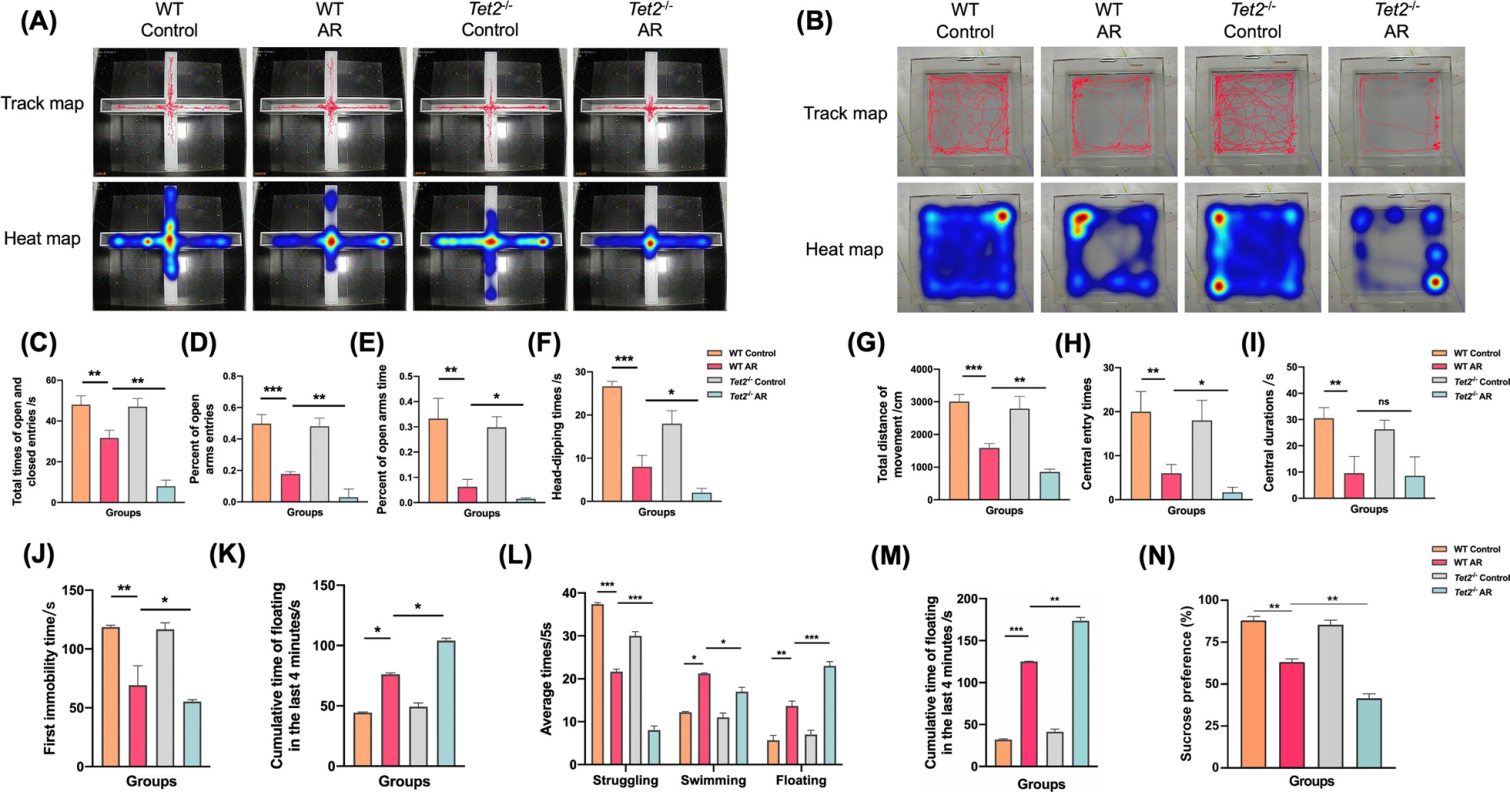
Behavioral experiments showed that TET2 deficiency aggravated anxiety and depression-like behaviors in AR mice. **A** Representative travel tracing in elevated cross maze test from mice. **B** Total time of open and closed entries. **C** Percent of open arms entries. **D** Percent of open arms times. **E** Head-dipping times. **F** Representative travel tracing in open field test from mice. **G** Total movement distance of mice. **H** Central entry times of mice. **I** Central durations of mice. **J** The first immobility time on tail suspension test. **K** Immobility time in the last 4 min on tail suspension test. **L** Average time spent in struggling behavior, swimming behavior, and floating behavior on the forced swim test. **M** Immobility time in the last 4 min on the forced swim test. **N** The sucrose preference (%) of sucrose preference test. (*P < 0.05, **P < 0.01, ***P < 0.001)

Compared with the control group, the total number of arm approaches in the AR group was significantly decreased, reflecting the decreased motor activity. The proportion of times entering the open arm and the proportion of time staying in the open arm decreased significantly, which reflected the aggravation of anxiety symptoms. The number of downward exploration decreased significantly, reflecting the increased fear and decreased exploration desire of AR mice. There was no significant difference between *Tet2*^−/−^ control group and control group. Compared with the AR group, the four data of the *Tet2*^−/−^ AR group were also significantly different, reflecting the further aggravation of corresponding symptoms, indicating that TET2 was involved in the anxiety-like behavior of AR mice, and the absence of TET2 would lead to the aggravation of anxiety-like behavior of AR mice (Fig. 2A, C–F).

#### Open field experiment

The open field experiment was used to detect the anxiety and depression-like behaviors of mice in Control, AR, *Tet2*^−/−^ control and *Tet2*^−/−^ AR groups. It was found that there were significant differences in the total distance of movement and the number of central zone entry among the four groups. Compared with the control group, the total movement distance, the number of central zone entry and the central zone residence time of AR mice were significantly decreased, reflecting the significant aggravation of anxiety and depression. There was no significant difference between *Tet2*^−/−^ control group and control group. Compared with the AR group, the total distance of movement and the number of central zone entry in the *Tet2*^−/−^ AR group were significantly decreased, reflecting more severe symptoms of anxiety and depression, and there was no significant difference in the residence time of central zone, indicating that TET2 was involved in the anxiety and depression-like behaviors of AR mice, and the absence of TET2 would lead to the aggravation of anxiety and depression-like behaviors of AR mice (Fig. 2B, G–I).

#### Tail suspension test

Compared with the control group, the first immobility time of the AR group was significantly decreased in the first 2 min, indicating that the AR group was more likely to induce depression. The cumulative immobility time of the last 4 min increased significantly, reflecting that the mice in the AR group were more prone to depression. There was no significant difference between Tet2−/− control group and control group. Compared with the AR group, the data of the Tet2−/−AR group were also significantly different, reflecting that the corresponding depressive symptoms were further aggravated, indicating that Tet2 was involved in the depression-like behavior of AR mice, and the absence of Tet2 would lead to the aggravation of depression-like behavior of AR mice (Fig. 2J, K).

#### Forced swimming test

Compared with the control group, the struggling times of the AR group were significantly decreased, while the swimming and floating times and the cumulative immobile time of the last 4 min were significantly increased, which reflected that the AR group was more likely to give up and the depressive symptoms were significantly aggravated. There was no significant difference between *Tet2*^−/−^ control group and control group. Compared with the AR group, the data of the four groups of mice in the *Tet2*^−/−^ AR group were also significantly different, reflecting the further aggravation of depressive symptoms, indicating that TET2 is involved in the depression-like behavior of AR mice, and the absence of TET2 will lead to the aggravation of depression-like behavior of AR mice (Fig. 2L, M).

#### Sucrose preference test

Compared with the control group, the sugar water preference rates of the AR group were significantly decreased. There was no significant difference between Tet2^−/−^ control group and control group. In addition, the sugar water preference rates was further reduced in the Tet2^−/−^ AR group compared with the wild type AR group, indicating that Tet2 was involved in the depression-like behavior of AR mice (Fig. 2N).

### TET2 depletion increases the metabolic level in ACC of AR mice

**Fig. 3:**
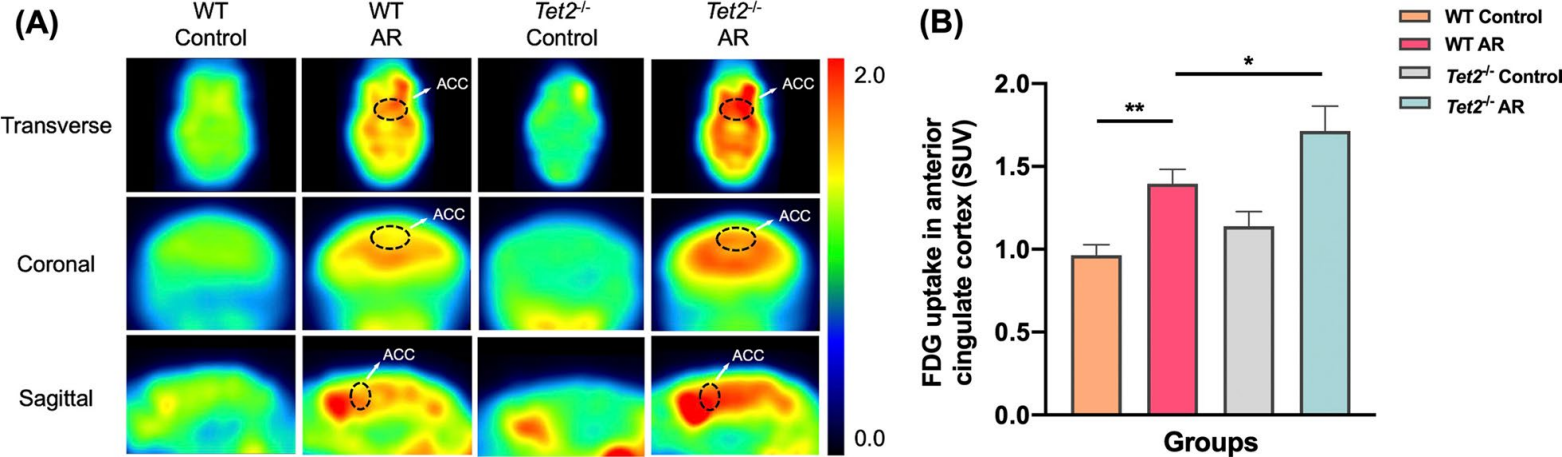
TET2 depletion increases the metabolic level in ACC of AR mice. **A** Representative transverse, coronal, and sagittal 18FDG-PET images of mouse head. **B** Quantitative analysis of mean FDG uptake in the ACC. (*P < 0.05, **P < 0.01)

In order to confirm the changes of ACC in AR mice, ^18^F-FDG PET experiments were performed on control, AR, *Tet2*^−/−^ control and *Tet2*^−/−^ AR mice, respectively. The mean standardized uptake value (SUV) was calculated by formula. Compared with the control group, the SUV values of ^18^F-FDG PET experiment in the ACC of AR group were significantly increased at the horizontal, coronal and sagittal levels. There was no significant difference in SUV value between *Tet2*^−/−^ control group and Control group. Compared with AR group, SUV value of mice in *Tet2*^−/−^ AR group was further increased. These results suggested that ^18^F-FDG was increased in the ACC of AR mice, and TET2 deletion further significantly increased the metabolic level of the ACC of AR mice (Fig. 3A, B).

### TET2 depletion aggravates nerve injury in ACC of AR mice

**Fig. 4:**
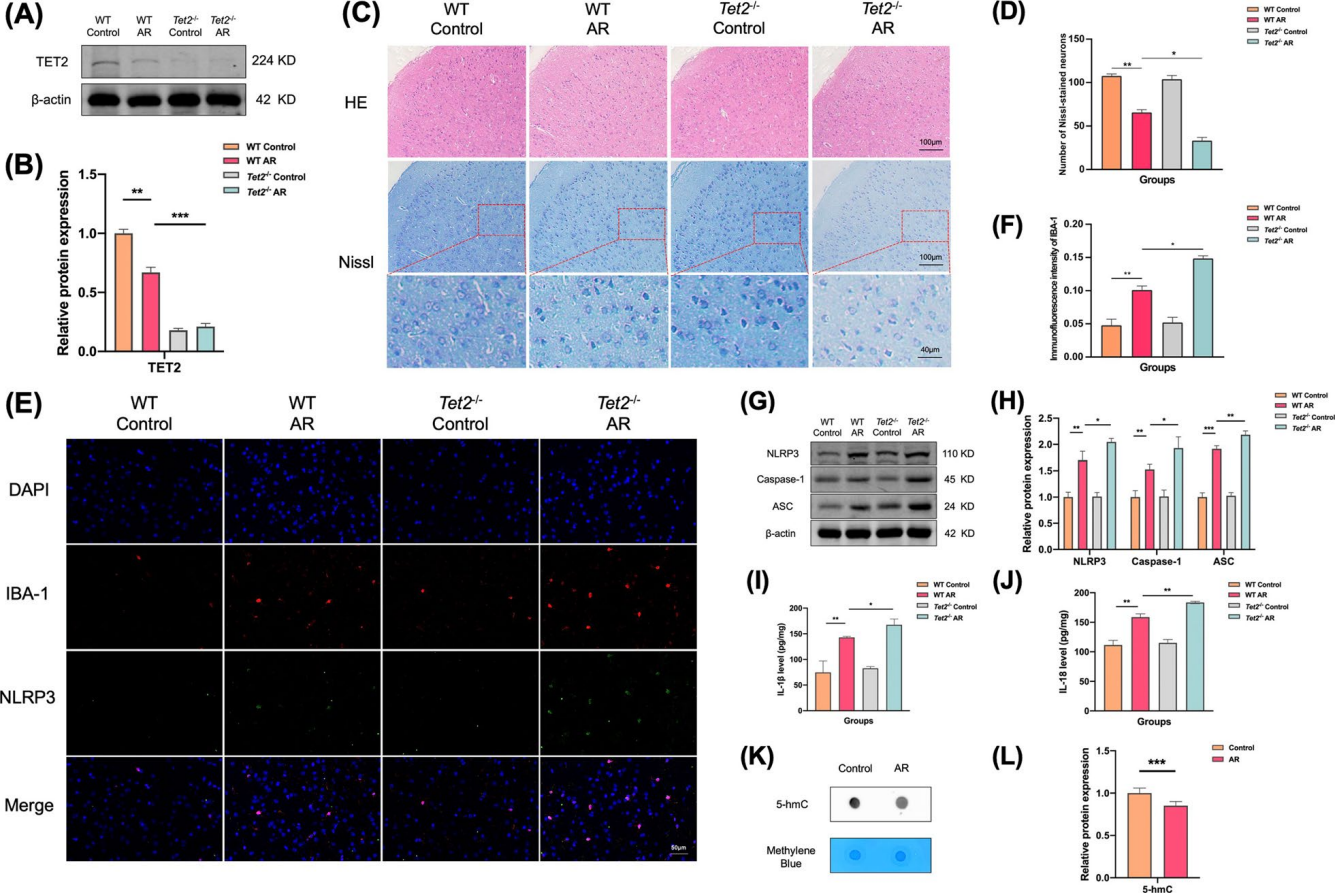
TET2 depletion aggravates nerve injury in ACC of AR mice. **A** Expression levels of TET2 in the ACC were determined using western blot analysis. **B** Quantitative analysis of band intensity. **C** Representative HE-stained and Nissl-stained images of the mice ACC. **D** Number of Nissl-stained neurons. **E** Representative immunofluorescence images of colocalization of NLRP3 in microglia in ACC. **F** Immunofluorescence of intensity of IBA-1. **G** Expression levels of NLRP3, Caspase-1, and ASC in the ACC were determined using western blot analysis. **H** Quantitative analysis of band intensity. **I**, **J** Levels of IL-1β and IL-18 were determined by ELISA in the ACC. **K** Levels of 5-hmC determined by dot blot in the ACC. **L** Quantitative analysis of 5-hmC levels in the ACC. Data are presented as mean ± SEM. (*P < 0.05, **P < 0.01, ***P < 0.001)

The expression level of TET2 protein in the ACC of Control, AR, *Tet2*^−/−^ control and *Tet2*^−/−^ AR groups was detected by Western Blot. The results showed that compared with the control group, the content of TET2 in the ACC of mice in the AR group was significantly decreased, and TET2 was not expressed in the ACC of mice in the *Tet2*^−/−^ control and *Tet2*^−/−^ AR groups, and the difference was statistically significant. This result indicates that TET2 is involved in the pathological process of AR in mice, and the expression of TET2 in the ACC of AR mice is significantly decreased compared with that of normal controls (Fig. 4A, B).

The results of HE staining showed that there were no significant structural changes and no significant differences in the overall cell number among the four groups of mice. The results of Nissl staining showed that compared with the control group, the number of Nissl bodies staining around the nucleus of the ACC of mice in the AR group was attenuated and the overall number of Nissl bodies was decreased. There was no significant difference between *Tet2*^−/−^ control group and control group. Compared with the AR group, Nissl bodies in the ACC of mice in the *Tet2*^−/−^ AR group were further reduced and attenuated (Fig. 4C, D).

Compared with the control group, more microglia were activated in the ACC of mice in the AR group, and more NLRP3 was expressed in the inflammasome. Colocalization results also showed that most of the inflammasome NLRP3 was highly expressed in microglia. There was no significant difference between *Tet2*^−/−^ control group and control group. Compared with the AR group, the corresponding expression was further increased in the ACC of mice in the *Tet2*^−/−^ AR group (Fig. 4E, F).

The WB (Fig. 4G, H) and ELISA (Fig. 4I, J) results showed that compared with the control group, the expression levels of NLRP3, Caspase-1, ASC, IL-1β and IL-18 in the ACC of AR group were increased. There was no significant difference in the expression of *Tet2*^−/−^ control group and control group. Compared with the AR group, the corresponding expression in the ACC of mice in the *Tet2*^−/−^ AR group was further increased.

### AR mice showed decreased levels of hydroxymethylation in ACC

TET protein is a dioxygenase, and TET2 is a member of the TET family. To further demonstrate from the epigenetic level that the decrease of TET2 level in the ACC region of AR mice aggravates AR pathology and anxiety and depression-like behaviors, we detected the expression of 5-hmC by Dot Blot. The results showed that the level of 5-hmC in the ACC of AR group was significantly lower than that of control group (Fig. 4K, L).

### TET2 affects NLRP3/IL-1β pathway activation in mouse microglia cell lines

We further explored the role of TET2-mediated NLRP3/IL-1β pathway in mouse microglia activation by in vitro cell experiments. Since OVA cannot cross the blood–brain barrier, it is speculated that the central neuroinflammation is not directly caused by OVA, but by inflammatory cytokines. Therefore, LPS was used to stimulate the mouse microglia cell line BV2 to simulate the central neuroinflammation in the ACC of AR mice after modeling, and TET2 was intervened. To observe the direct effect of TET2 on the activation state of microglia, and the changes of NLRP3/IL-1β inflammasome pathway after TET2 up-regulation or down-regulation.

**Fig. 5:**
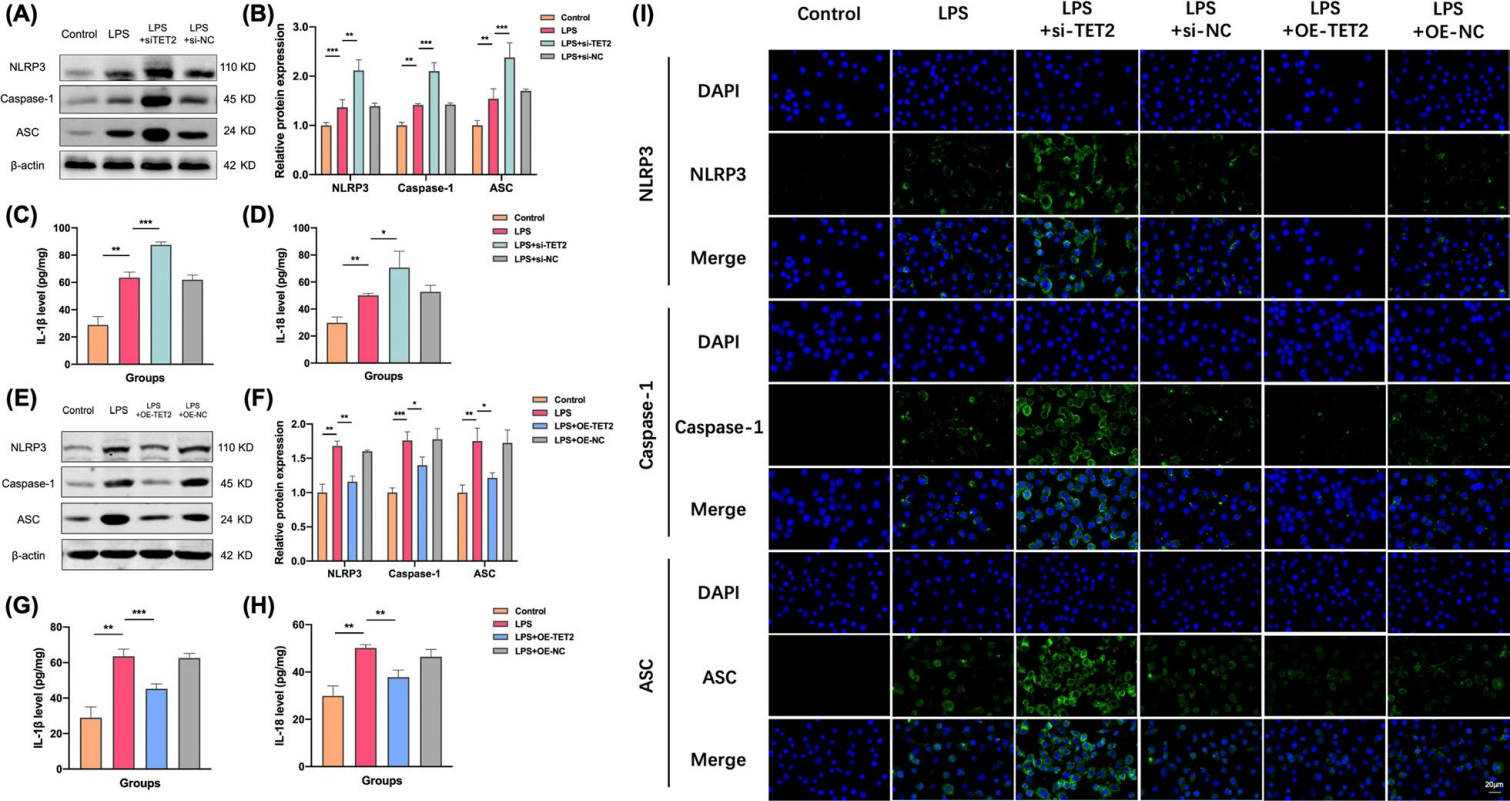
TET2 affects NLRP3/IL-1β pathway activation in mouse microglia cell lines. **A** Representative western blot analysis of TET2, NLRP3, Caspase-1, and ASC protein in the BV-2 cells from three groups. **B** Normalized expression of TET2, NLRP3, Caspase-1, and ASC protein in the BV-2 cells by western blot analysis. **C**, **D** Levels of IL-1β and IL-18 were determined by ELISA in the cell culture supernatant. **E** Representative western blot analysis of TET2, NLRP3, Caspase-1, and ASC protein in the BV-2 cells from three groups. **F** Normalized expression of TET2, NLRP3, Caspase-1, and ASC protein in the BV-2 cells by western blot analysis. **G**, **H** Levels of IL-1β and IL-18 were determined by ELISA in the cell culture supernatant. **I** Representative immunofluorescence images of colocalization of NLRP3, Caspase-1, and ASC in the BV-2 cells from three groups. Data are presented as mean ± SEM. (*P < 0.05, **P < 0.01, ***P < 0.001)

Firstly, microglial cell lines were stimulated with LPS at a concentration of 1 μg/mL to observe the morphological changes of microglial cells. Then, TET2 knockdown was transfected to intervene the above pathways, and the changes of the downstream NLRP3/IL-1β inflammasome pathway were observed by Western Blot and ELISA. The results showed that compared with the control group, the expression levels of NLRP3, Caspase-1, ASC, IL-1β and IL-18 in microglia in LPS group were increased (Fig. 5A–D). After TET2 knockdown, the expression of corresponding indexes in each group was further increased. After overexpression of TET2, the expressions of NLRP3, Caspase-1, ASC, IL-1β and IL-18 in microglia of LPS OE-TET2 group were significantly decreased compared with LPS group (Fig. 5E–H).

Immunofluorescence staining results showed that compared with the control group, the expression of NLRP3, Caspase-1 and ASC in microglia in LPS group were increased. After knockdown of TET2, the expression of inflammasome was further significantly increased. After overexpression of TET2 on the basis of LPS model, NLRP3, Caspase-1 and ASC expression of microglia were significantly decreased (Fig. 5I).

### Metformin alleviates anxiety-depression-like behaviors in AR mice

**Fig. 6:**
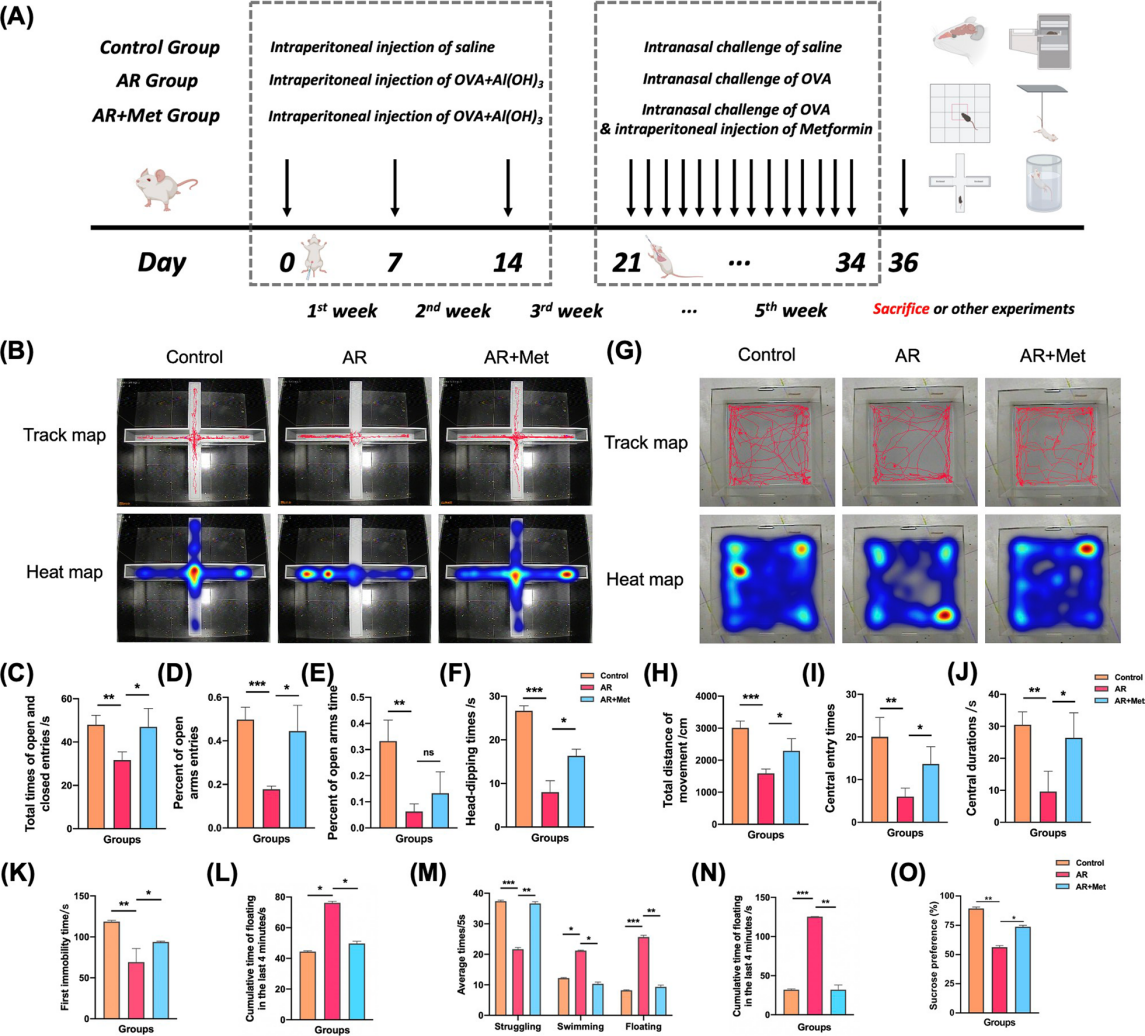
Effect of metformin on alleviating nasal inflammation and symptoms and anxiety and depression-like behaviors in AR mice. **A** Schematic timeline of the experimental procedure. **B** Representative travel tracing in elevated cross maze test from mice. **C** Total time of open and closed entries. **D** Percent of open arms entries. **E** Percent of open arms times. **F** Head-dipping times. **G** Representative travel tracing in open field test from mice. **H** Total movement distance of mice. **I** Central entry times of mice. **J** Central durations of mice. **K** The first immobility time on tail suspension test. **L** Immobility time in the last 4 min on tail suspension test. **M** Average time of struggling behavior, swimming behavior, and floating behavior on the forced swim test. **N** Immobility time in the last 4 min on the forced swim test. **O** The sucrose preference (%) of sucrose preference test. (*P < 0.05, **P < 0.01, ***P < 0.001)

In addition to the control group and AR group, mice in the AR + Met group were intraperitoneally injected with metformin (250 mg/kg) 30 min before each stimulation stage after basic sensitization (Fig. 6A). In the cell experiment, LPS was used to model neuroinflammation in mouse microglia cell line BV2, and the central nervous inflammation in the ACC region was simulated after AR mouse model. TET2 was interfered and metformin (2 mM) was given to observe the changes of NLRP3/IL-1β pathway in microglia cell line BV2. Through elevated cross maze, open field, tail suspension, force swimming and sucrose preference experiments, we found that metformin alleviated anxiety and depression-like behaviors in AR mice (Fig. 6B–O).

### Metformin reduces the metabolism level of the ACC and reduces the nerve injury of the ACC in AR mice

**Fig. 7:**
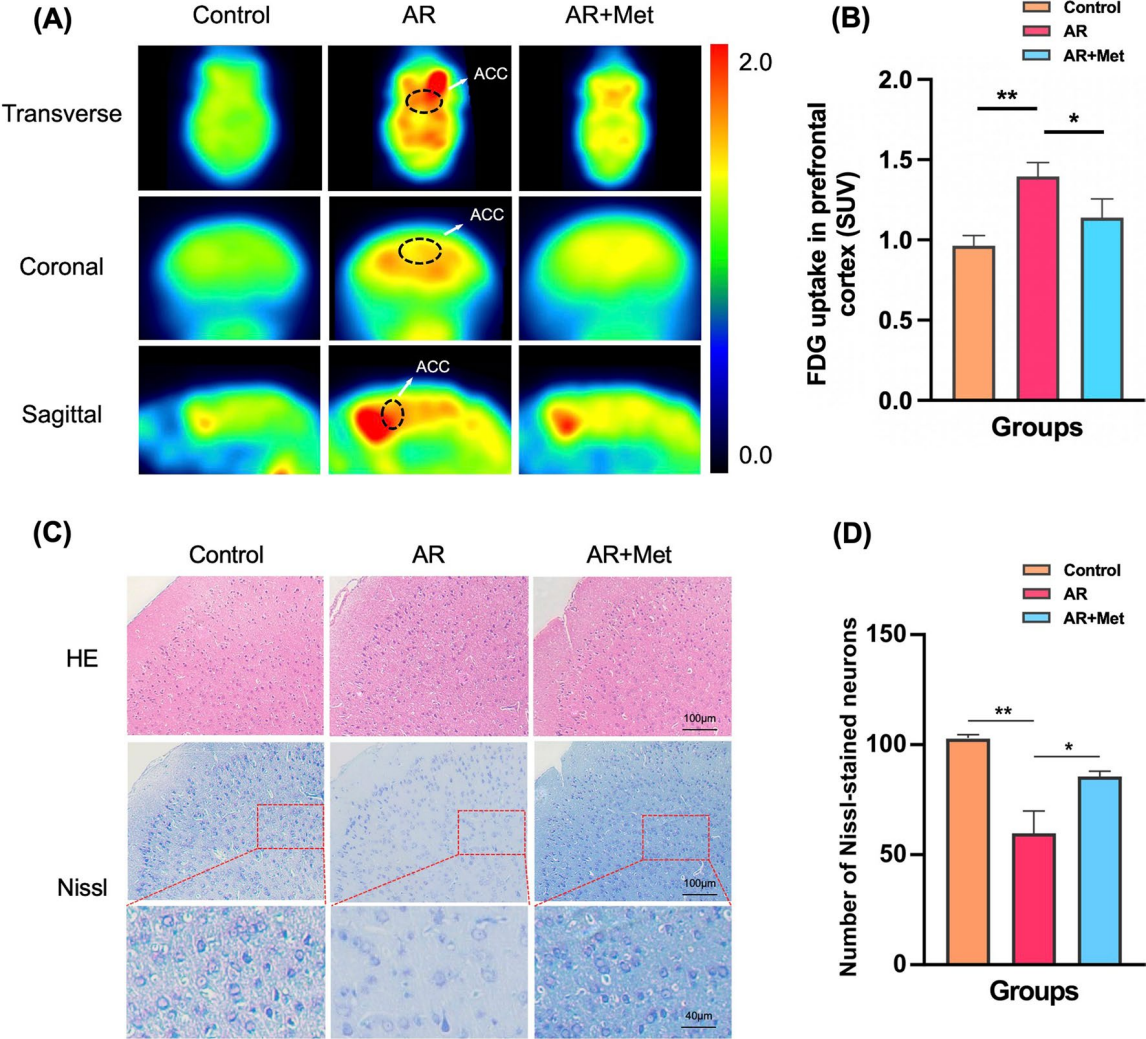
Metformin reduces the metabolic level and the nerve injury of ACC in AR mice. **A** Representative transverse, coronal, and sagittal 18FDG-PET images of mouse head. **B** Quantitative analysis of mean FDG uptake in the ACC. **C** Representative HE-stained and Nissl-stained images of the mice ACC. **D** Number of Nissl-stained neurons. Data are presented as mean ± SEM. (*P < 0.05, **P < 0.01)

To explore the effects of metformin on the metabolic level and inflammatory damage of ACC, a brain area related to anxiety and depression-like behavior in AR mice, we performed ^18^F-FDG PET experiments in Control group, AR group and AR + Met group. The mean standardized uptake value (SUV) was calculated by formula. Compared with the AR group, the SUV value of the AR + Met group mice decreased significantly. The results showed that metformin could significantly reduce ^18^F-FDG uptake rate in the ACC of AR mice, that is, significantly reduce the metabolic level of the ACC (Fig. 7A, B).

HE staining showed no significant structural changes among the three groups of mice. The results of Nissl staining showed that compared with the control group, the staining of Nissl bodies around the nucleus of the ACC area of the AR group was lighter, and the overall number was reduced. Compared with the AR group, the staining of Nissl bodies in the ACC of the AR + Met group was darker and the overall number was increased (Fig. 7C, D).

### Metformin affects the activation of NLRP3/IL-1β pathway in mouse microglia cell lines

**Fig. 8:**
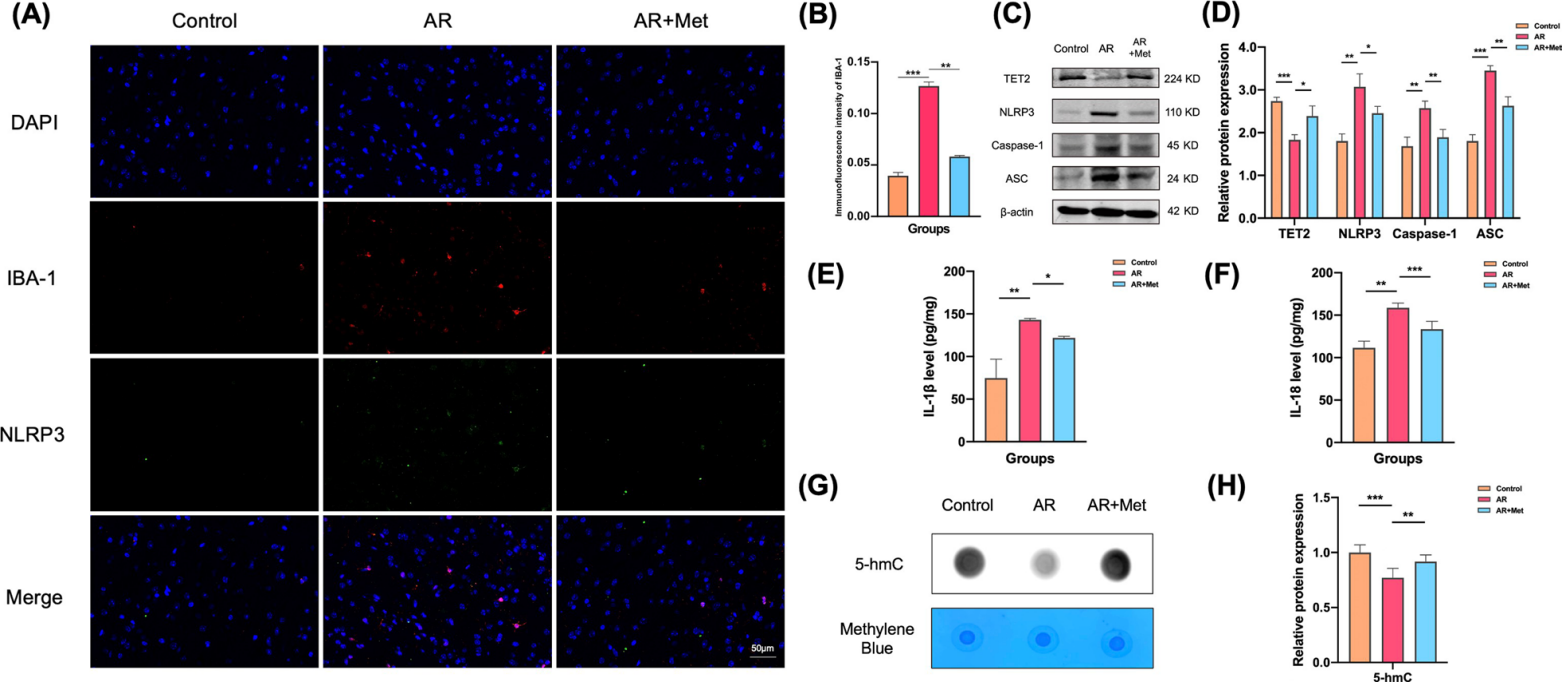
Metformin inhibits microglial activation in ACC of AR mice through NLRP3/IL-1β pathway. **A** Representative immunofluorescence images of colocalization of NLRP3 in microglia in ACC. **B** Immunofluorescence of intensity of IBA-1. **C** Representative western blot analysis of TET2, NLRP3, Caspase-1, and ASC protein in the ACC from three groups. **D** Normalized expression of TET2, NLRP3, Caspase-1, and ASC protein in the ACC by western blot analysis. **E**, **F** Levels of IL-1β and IL-18 were determined by ELISA in the ACC. **G** Levels of 5-hmC determined by dot blot in the ACC. **H** Quantitative analysis of 5-hmC levels in the ACC from three groups. Data are presented as mean ± SEM. (*P < 0.05, **P < 0.01, ***P < 0.001)

Double fluorescence staining of microglia marker proteins IBA-1 and NLRP3 was also performed in the AR + Met group. The results showed that compared with the control group, more microglia were activated in the ACC of the AR group, and more inflammasome NLRP3 was expressed. The colocalization results also showed that most of the inflammasome NLRP3 was highly expressed in microglia. Compared with the AR group, the corresponding expression in the ACC of the AR + Met group was significantly reduced (Fig. 8A, B). WB and ELISA results showed that compared with the AR group, the expression of TET2 in the ACC of the AR + Met group was significantly increased, and the expression of NLRP3, caspase-1, ASC, IL-1β and IL-18 was significantly decreased (Fig. 8C–F). The expression of 5-hmC was determined by Dot Blot technique. The results showed that the level of 5-hmC in the ACC of AR + Met group was significantly higher than that of AR group (Fig. 8G, H).

**Fig. 9:**
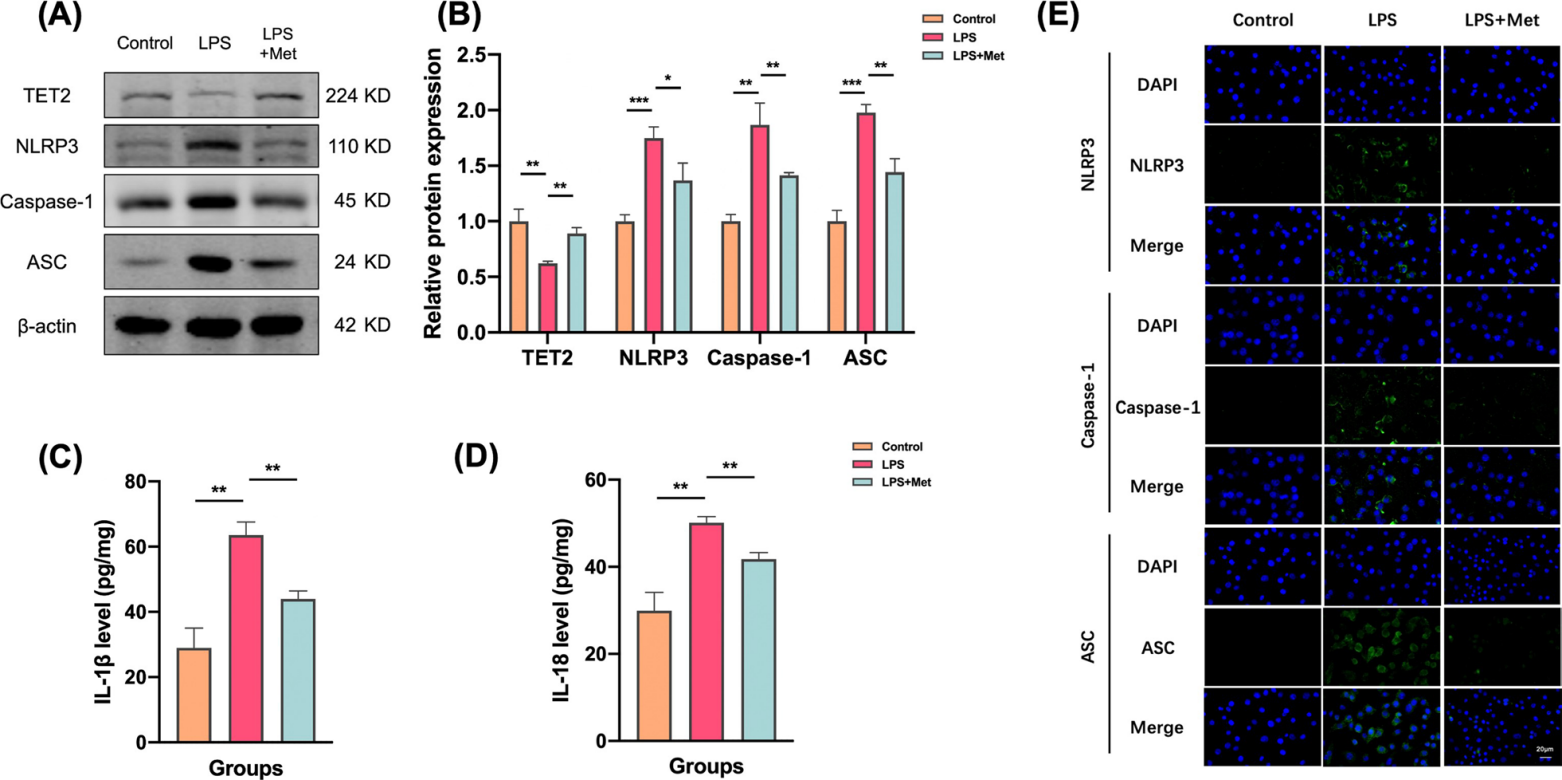
Metformin inhibits NLRP3/IL-1β pathway activation through TET2 in microglia cell lines. **A** Representative western blot analysis of TET2, NLRP3, Caspase-1, and ASC protein in the BV-2 cells from three groups. **B** Normalized expression of TET2, NLRP3, Caspase-1, and ASC protein in the BV-2 cells by western blot analysis. **C**, **D** Levels of IL-1β and IL-18 were determined by ELISA in the cell culture supernatant. **E** Representative immunofluorescence images of colocalization of NLRP3, Caspase-1, and ASC in in the BV-2 cells from three groups. Data are presented as mean ± SEM. (*P < 0.05, **P < 0.01, ***P < 0.001)

In order to further verify the role of metformin in the activation of mouse microglia through TeT2-mediated NLRP3/IL-1β pathway, the mouse microglia cell line BV2 was used to establish a model of neuroinflammation, simulate the central neuroinflammation state in the posterior ACC of AR mice, and intervene TET2. Metformin (2 mM) was given to observe the changes of NLRP3/IL-1β pathway in microglia cell lines. First, the mouse microglial cell line was stimulated with LPS at a concentration of 1 μg/mL to observe the microglial state. Then, one group was added with metformin (LPS + Met group), and the downstream NLRP3/IL-1β pathway was observed by Western Blot and ELISA. The results showed that compared with the control group, the expression levels of NLRP3, caspase-1, ASC, Il-1β and IL-18 in microglia in the LPS group were increased. Compared with LPS group, the expression of corresponding indexes in LPS + Met group was significantly decreased (Fig. 9A–D). The results of immunofluorescence staining showed that compared with the control group, the expressions of NLRP3, caspase-1 and ASC in microglia in the LPS group were increased. After adding metformin on the basis of LPS model, the expression of NLRP3, caspase-1 and ASC in microglia was significantly decreased (Fig. 9E).

## Discussion

AR is thought to be associated with mood disorders, particularly anxiety and depression. A cross-sectional study demonstrated that the frequency of anxiety and depression in the AR group was 45.9% and 35.7%, respectively, compared with 10.4% and 16.6% in the control group (Bedolla-Barajas et al. 2017). The occurrence of anxiety and depression is common in AR patients. They also found that women were more likely to suffer from anxiety and depression. In addition, Wang et al. compared the psychological status of 222 AR patients and 133 healthy controls by means of a questionnaire and showed that anxiety and depression were prevalent in AR patients and that severity of disease and low education were risk factors for depression and anxiety (Wang et al. 2022). A recently published Meta-analysis by Rodrigues et al. showed that AR was related to a high risk of anxiety and depression (Rodrigues et al. 2021). Our study also showed results that were consistent with previous studies. Behavioral experiments showed that mice in the AR group exhibited significant anxiety- and depression-like behavior compared to normal controls. Therefore, an in-depth exploration of the mechanisms of anxiety and depression in AR is necessary.

The ACC is a key brain region responsible for the processing of negative emotions and is thought to be closely associated with anxiety and depression (Hare and Duman 2020). Alterations in the neuronal activity and morphology and of the ACC are closely associated with depression disorders (Xu et al. 2019a). Reduced apical dendritic branching and basilar and of the ACC neurons were observed in mice with depression (Nashed et al. 2015). Additionally, functional magnetic resonance imaging (fMRI) revealed abnormal functional connectivity and shifted activity of ACC in patients with depression (Xu et al. 2019b). Anxiety disorders are also associated with changes in synaptic plasticity, neuronal activity, and connectivity in the ACC (Xu et al. 2019a). There is evidence that patients suffering from anxiety disorders are significantly reduced in anxiety rating after being treated with 1 Hz repetitive transcranial magnetic stimulation in the ACC (Herrmann et al. 2017). A wide range of neurotransmitter systems within the ACC play an important role in anxiety disorder pathophysiology, including cholinergic, serotoninergic glutamatergic, and GABAergic, systems (Bukalo et al. 2014). Recently developed optogenetic and chemogenetic methods have preliminarily elucidated the important contribution of the ACC and its circuitry to anxiety and depression. Impairment of olfactory bulb-medial prefrontal cortex circuits has been shown to be potentially important in inducing anxiety-like behavior in experimental models of AR (Salimi et al. 2019). A recent neuroimaging study by our team showed that changes in the ACC activity in AR patients correlate with their anxiety and depression symptoms (Gao et al. 2021). All of this evidence suggests that the ACC may be a key brain region associated with anxiety and depression in AR.

Microglia are important immune cells in the central nervous system (CNS) and have a key role in maintaining CNS homeostasis. There is substantial evidence that neuroinflammation caused by abnormal microglia activation in emotion-related brain regions, such as the ACC and hippocampus, promotes the development of anxiety and depression (Wohleb and Delpech 2017). In the CNS, the NLRP3 inflammasome is mainly expressed in microglia and is considered to be a major contributor to neuroinflammation. Wu et al. reported that expression levels of NLRP3 and IL-18 in microglia correlated with lipopolysaccharide (LPS) challenge-induced anxiety and depressive-like behaviors in mice (Wu et al. 2021). In addition, Yamanashi et al. found that beta-hydroxybutyrate, an endogenic NLRP3 inflammasome inhibitor, can alleviate anxiety and depressive-like symptoms induced by chronic unpredictable stress (CUS) (Yamanashi et al. 2017). Our results also show significant microglia activation in the prefrontal cortex of OVA-exposed mice, accompanied by increased expression of NLRP3 and IL-1β. Based on our present study and the prior literature, we hypothesized the NLRP3 inflammasome activation of microglia in the prefrontal cortex, which promotes anxiety and depressive symptoms in AR. Several studies have shown that OVA-induced allergic disease models can lead to the development of neuroinflammation in the brain. OVA is a high molecular weight protein with a molecular weight of 45 kDa. However, the blood–brain-barrier (BBB) limits the uptake of molecules greater than 500 Da into the CNS. Therefore, it is unlikely that the abnormal alterations in the ACC in AR mice are directly caused by OVA. Combined with previous studies, we hypothesize that the microglial inflammation observed herein may be closely related to peripheral inflammatory factors crossing the BBB to exert their effects, or other mechanisms (Saitoh et al. 2021; Tamayo, et al. 2022), which remain to be explored.

Epigenetic modifications play an important role in the development and maintenance of microglia-mediated neuroinflammation. As of now, TET proteins are well recognized as epigenetic master regulators capable of establishing dynamic local and overall methylation landscapes through various pathways, intimately involved in a variety of fundamental biological processes (Kohli and Zhang 2013). Compared to other members of the TET family, TET2 is most closely associated with inflammation and immunity. In a study published in Science, Fuster et al. first revealed a link between TET2 inactivation and the NLRP3/IL-1β pathway (Fuster et al. 2017). They found that TET2 plays an important role as a "brake" for NLRP3 inflammasome activation in peripheral macrophages. Tet2-deficient macrophages exhibit strong IL-1β secretion capacity and promote the formation and development of atherosclerosis in mice (Fuster et al. 2017). Additionally, studies have shown that microglia-dependent neuroinflammation is associated with epigenetic modification of TET2. For example, *Tet2* specific knockout in the hippocampus promotes microglial activation and exacerbates cognitive dysfunction in Alzheimer's disease mice (Li et al. 2020). In this part of the experiment, we found that TET2 and DNA 5-hmC levels were down-regulated in the ACC of AR mice, and the NLRP3/IL-1β pathway protein in microglia was further increased in *Tet2*^*−/−*^ AR mice compared with WT AR mice. After knocking down TET2 in mouse microglia cell line BV2, NLRP3 inflammasome-related proteins were significantly increased, suggesting that the down-regulation of TET2 is closely related to the activation of NLRP3/IL-1β pathway in microglia. Therefore, TET2 affects the activation of NLRP3/IL-1β pathway in microglia, which plays an important role in the occurrence and development of AR anxiety and depression-like behavior.

Metformin, a biguanide, is one of the first-line medications for Type 2 Diabetes Mellitus (T2DM) (Sanchez-Rangel and Inzucchi 2017). In addition to its antihyperglycemic efficacy, metformin has also been shown to rapidly cross the blood–brain barrier and directly affect the central nervous system, thereby reducing neuroinflammation (Madhu et al. 2022). It has been demonstrated that metformin can be beneficial in experimental models of neuroinflammatory diseases. Metformin exerts anti-inflammatory effects by inhibiting multiple signaling pathways such as ERK1/2, PI3K/AKT/NF-κB, and JAK/STAT in microglia to reduce the activate inflammatory cascades (Khezri et al. 2022). A Danish clinical study showed that metformin has a positive effect on the treatment of depression and is recommended for diabetic patients at risk of depression (Kessing et al. 2020). The effects of metformin on depression-like behavior were observed in rats following oxandrolone administration by modulating the production of pro-inflammatory cytokines, including IL-1β, IL-6, and TNF-α (Hammad et al. 2021). In addition, metformin inhibits activation of NF-κB-NLRP3 inflammation in hippocampus of mice, thereby alleviating depressive-like symptoms (Du and Bu 2020). Metformin ameliorates ethanol-induced anxiety-like behavior in rats by reducing oxidative stress in the frontal cortex and hippocampus (Bonea et al. 2020). In line with previous studies, our results also show that metformin has good anxiolytic and antidepressant effects, which may be attributed to its inhibition of microglia inflammation.

From the perspective of epigenetics, metformin has also attracted attention in recent years by acting on TET2 to affect the occurrence and development of diseases. Studies have shown that metformin plays an antidepressant role by increasing the expression of brain-derived neurotrophic factor BDNF through the TET2-related pathway (Wang et al. 2019). In vascular smooth muscle cell (VSMC) dysfunction mediated by type 2 diabetes conditions, studies have shown that metformin restores TET2 by inhibiting the expression of YTHDC2 and circYTHDC2, thereby preventing the progression of VSMC dysfunction under high glucose conditions (Yuan et al. 2021). In the field of oncology, an article in the journal Science found that metformin treatment protected phosphorylation at serine 99, thereby improving TET2 stability and 5-hmC levels, which in turn stabilized tumor suppressor genes, revealing an epigenetic pathway for metformin-mediated tumor suppression (Wu et al. 2018). In transcriptional subtyping studies of pancreatic ductal adenocarcinoma, TET2-driven 5-hmC signature of GATA6 was confirmed, and the use of metformin can enhance TET2 stability and subsequently restore 5-hmC and GATA6 levels, restoring the squamoid tumor phenotype and Wnt dependence in vitro and in vivo (Eyres et al. 2021). In the study of head and neck squamous cell carcinoma (HNSCC), metformin can also inhibit cell proliferation and migration by restoring the expression of the tumor suppressor gene *Tet2* (Huang et al. 2020). Thus, the research that metformin mediates 5-hmC hydroxymethylation by restoring TET2 expression to improve the disease process has been confirmed in the field of epigenetics. Our study also confirmed the effect of metformin on the level of hydroxymethylation in the ACC of AR mice from an epigenetic perspective, and then improved AR pathology and anxiety and depression-like behaviors.

Overall, although there is some evidence for the relationship between allergic rhinitis and brain-related symptoms, the mechanism is still unclear. It is speculated that the pathogenesis may have the following aspects: (1) physiological effects. The physiological effects of nasal obstruction and its impairing effect on sleep may together contribute to the production of negative psychiatric symptoms. (2) Cytokines. Proinflammatory cytokines can enter the central nervous system (CNS) and interact with the cytokine network in the brain, possibly affecting various aspects of brain-related behavior through different pathways. (3) Neuroinflammation. Microglia and astrocytes are involved in the initiation of pro-and anti-inflammatory events and may induce degeneration of neurons. (4) Inheritance. There may be a shared genetic risk between allergic diseases and depression.

Limitations of this study include that: in animal experiments, the mice we used are gene knockout mice. Although there was no significant difference in behavior and other indicators between *Tet2*^*−/−*^ mice and control mice, it is undeniable that the conditional knockout mice can better explore the role and molecular mechanism of genes or proteins in vivo. Another limitation is that although LPS modeling method in cell experiments is recognized as a better scheme in neuroinflammation research, it cannot completely represent the environment of human or mouse central nervous system in AR state. Also, we did not observe the expression changes of TET2 by interfering with NLRP3. In addition, both in vitro and in vivo data of this study cannot prove that metformin acts entirely through TET2 and subsequent studies can further explore the mechanism of metformin through knock-out mice or siRNA methods. The dose of metformin was also based on the reference in the literature, and the relationship between the concentration gradient and the efficacy was not explored. In the follow-up experiments, we will further optimize the experimental protocol and explore the mechanism of action, so as to provide a new perspective and scheme for the research and treatment of AR central neuroinflammation and anxiety and depression-like behavior.

## Conclusions

AR mice show anxiety and depression-like behaviors. The NLRP3/IL-1β pathway of microglia in the ACC of AR mice is involved in the pathological process of anxiety and depression-like behavior, and the loss of TET2 further activates the NLRP3/IL-1β pathway of microglia in AR mice. In addition, we found that metformin could regulate the NLRP3/IL-1β pathway by increasing the expression of TET2, thereby reducing the activation of microglia in the ACC and alleviating central neuroinflammation in AR mice. Our study is expected to explore new possibilities for the mechanism and treatment of AR central neuroinflammation.

## Data Availability

All data generated or analyzed during this study are included in this article.
